# Hardware-Assisted Security Monitoring Unit for Real-Time Ensuring Secure Instruction Execution and Data Processing in Embedded Systems

**DOI:** 10.3390/mi12121450

**Published:** 2021-11-26

**Authors:** Xiang Wang, Zhun Zhang, Qiang Hao, Dongdong Xu, Jiqing Wang, Haoyu Jia, Zhiyu Zhou

**Affiliations:** School of Electronic and Information Engineering, Beihang University, Beijing 100191, China; microzhun@buaa.edu.cn (Z.Z.); haoqiang1994@buaa.edu.cn (Q.H.); xudongdong1994@buaa.edu.cn (D.X.); wangjiqing@buaa.edu.cn (J.W.); sy1902109@buaa.edu.cn (H.J.); zhiyuzhou@buaa.edu.cn (Z.Z.)

**Keywords:** embedded system, security monitoring unit (SMU), system-on-chip (SoC), instruction monitoring, data monitoring

## Abstract

The hardware security of embedded systems is raising more and more concerns in numerous safety-critical applications, such as in the automotive, aerospace, avionic, and railway systems. Embedded systems are gaining popularity in these safety-sensitive sectors with high performance, low power, and great reliability, which are ideal control platforms for executing instruction operation and data processing. However, modern embedded systems are still exposing many potential hardware vulnerabilities to malicious attacks, including software-level and hardware-level attacks; these can cause program execution failure and confidential data leakage. For this reason, this paper presents a novel embedded system by integrating a hardware-assisted security monitoring unit (SMU), for achieving a reinforced system-on-chip (SoC) on ensuring program execution and data processing security. This architecture design was implemented and evaluated on a Xilinx Virtex-5 FPGA development board. Based on the evaluation of the SMU hardware implementation in terms of performance overhead, security capability, and resource consumption, the experimental results indicate that the SMU does not lead to a significant speed degradation to processor while executing different benchmarks, and its average performance overhead reduces to 2.18% on typical 8-KB I/D-Caches. Security capability evaluation confirms the monitoring effectiveness of SMU against both instruction and data tampering attacks. Meanwhile, the SoC satisfies a good balance between high-security and resource overhead.

## 1. Introduction

The modern embedded system integrates a reduced instruction set computing (RISC) processor core, specific functional modules, commonly-needed peripherals, and memory blocks on a single chip, for achieving the desired functions according to specific application requirements, such as increasing computing performance, keeping low power consumption, and improving reliability in the radiation environment [1,2,3]. The widespread applications of embedded systems are pushing systems-on-chip (SoCs) toward the dramatic improvements in performance and multifunction; however, these improvements are accompanied with the higher hardware complexity and various security threats [4]. Diversiform attacks can arise from the untrusted intellectual properties (IPs) [5], vulnerable hardware and software [6], and even insecure communication with remote devices [7], which are potential methods for jeopardizing the execution security of embedded systems in safety-critical applications. In practice, the various forms of existing and emerging attacks can be classified into the two main types: hardware-level attack and software-level attack. In hardware-level attacks, hardware Trojan is a typical example which could be inserted into an internal logic and activated under a specific condition to cause the processor unintended behaviors or program execution failures. Especially, recent reports show that attackers are inserting the hardware Trojans into memories to leak and modify critical data [8,9], which further aggravates the security concerns of SoCs in security-critical applications. The software-level attacks are mainly exploiting some vulnerabilities or bugs in programs to disturb instruction executions or perform other unintended actions, such as tampering program code or data and injecting malicious code. At present, most of embedded programs are written in the high-level programming languages of C and C++, which can access memory directly without any valid bound checks. These software-level attacks make it easy to implement the buffer overflow [10,11] via stack smashing and take control of the hardware platform during executing untrusted programs.

Function failure and data leakage have been emerging as the primary manifestations of being attacked in processing-intensive platforms, where the security of instruction execution and data processing in embedded systems must be guaranteed. An intelligent attacker can exploit the modified instruction and tampered data to trick the processor core’s internal interpreter into executing unintended instructions and accessing unauthorized data. Generally, for a trusted SoC chip, external memory Trojan and external physical attack are the two critical factors on damaging the trustworthiness of embedded systems. For example, the hardware Trojan could be designed and inserted into external flash memory to change program codes to cause the unintended behaviors or execution failures. In addition, external physical attacks could exploit high-tech detection instruments to implement bus monitoring and offline analysis for obtaining confidential data [12] and inject tampered data to disorganize program intended behaviors through external access interfaces.

Several security mechanisms have been proposed to protect the program executions against the potential hardware Trojans. Existing techniques by using standard functional validation [13,14] and side-channel analysis [15,16] after the chip manufactures can detect hidden hardware Trojans and do not need any hardware resource consumption. However, the high complexity of SoC with integrating several hundreds of IPs makes it expensive and time consuming to fully test and validate all the IP modules, and some purposefully inserted Trojans are developed to be exercised by rare events under a specific execution condition, so that they make it especially difficult to activate, analyze, and identify hardware Trojans in a functional validation environment. In particular, the commercial design of SoC architectures mainly adopts the efficient integration approach of IPs, and many IPs are acquired from some untrusted third-party vendors for shortening the time to market of applied products. This approach further increases the risk of malicious attacks. The side-channel analysis techniques can detect inserted Trojans by observing power consumption, circuit delay, electromagnetic emission and circuit noise at the postfabrication stage [17]. However, the effectiveness of side-channel analysis is greatly affected by the technology variation and highly depends on a golden reference design; therefore, that method is limited by the device parameter variations of nanometer technology.

Damaging program execution is not the only risk challenge for a trusted SoC chip; the off-chip main memory connecting with target SoC is also a vulnerable device, which can suffer from the external attacks of bus monitoring and offline analysis, and then, the adversaries can exploit the external data bus to launch physical tampering attacks with malicious data injections [18]. For example, the attackers can steal sensitive data during the dynamic data exchanges and exploit tampered data into memory stack and heap segments to change program executions out of the original intentions. Therefore, the confidentiality of dynamic data between the main memory and SoC is also an important concern for program execution security. In practice, few techniques were applied to encrypt the dynamic data of embedded systems in real time, since it was harder to predict dynamic data than instruction transfer during program execution without a significant speed degradation. To the best of our knowledge, program execution monitoring and data processing monitoring are mostly researched independently in existing techniques, and it is significantly necessary to provide a reinforced protection for both instruction execution and data processing security.

This paper presents a hardware-assisted security monitoring unit (SMU) for embedded system to provide real-time security monitoring and authenticated encryption. The integration of SMU can comprehensively prevent unintended instruction behaviors, dynamic data leakages, and physical tampering attacks caused by the off-chip threats. The specific contributions of this paper are summarized as follows:

- An instruction monitor is constructed in SMU to real-time monitor instruction executions, and any instruction tampering is detected by program basic block (BB) integrity checking mechanism;
- A data monitor is also constructed in SMU to complete the authenticated encryption and dynamic data monitoring for preventing dynamic data leakages and data tampering attacks, and any unauthorized change of ciphertext or signature in external main memory is detected by Tag integrity checking;
- The I-Cache, D-Cache, and monitor cache (M-Cache) are felicitously configured to significantly reduce the system performance overhead induced by SMU, and its average performance overhead reduces to as low as 2.18%;
- The experimental evaluations of security capability and hardware consumption confirm that the monitoring mechanism of SMU satisfies a good balance between high-security capability and low hardware complexity.

The remainder of this paper is organized as follows: Section 2 introduces SoC security assumptions and threat models considered in this work. Section 3 presents the related works concerning design strategies of instruction monitoring, information leakage, and physical data tampering. Section 4 and Section 5 present the hardware implementation mechanisms of instruction monitor and data monitor in SMU, respectively. Section 6 presents the experimental evaluations of SMU about performance overhead, security capability, and practicality comparison. The SoC hardware implementation is presented in Section 7. This paper is concluded in Section 8.

## 2. Security Assumptions and Threat Models

Before developing a hardware-assisted SMU for a target-embedded system platform, its specific security trustworthy assumption and threat model should first be determined, and the associated assumptions of all the design components (including IP entities) should be classified as trustworthy and untrustworthy. Hence, we make the following assumptions regarding the hardware architecture, hardware Trojan setting, and off-chip physical attacks.

**Figure 1:**
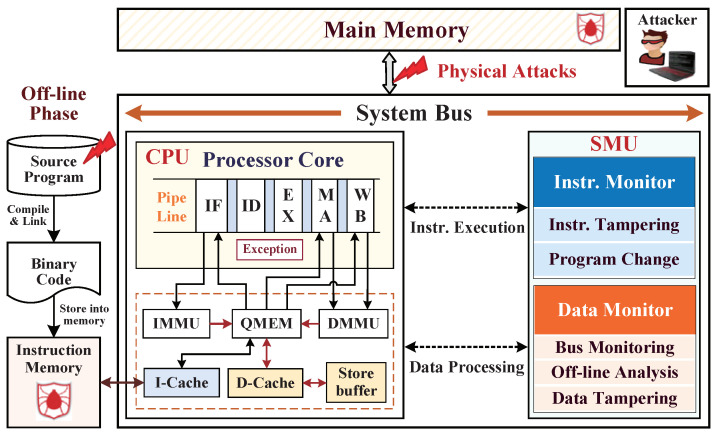
The SoC hardware architecture with integrating SMU for real-time security monitoring.

We propose an embedded system architecture based on the open-source reduced instruction set computing (OpenRISC) processor OR1200. This softcore processor is constructed with a Harvard microarchitecture; meanwhile, the sequential execution of central processing unit (CPU) consists of a five-stage pipeline of the instruction fetching (IF) stage, instruction decoding (ID) stage, instruction executing (EX) stage, memory accessing (MA) stage, and write back (WB) stage. The CPU connects with some commonly needed peripherals, such as the addressable quick memory (QMEM), instruction cache (I-Cache), data cache (D-Cache), and other components. We extended a proposed hardware-assisted SMU with CPU by the Wishbone bus to real-time monitor instruction execution and data processing. The SoC hardware architecture with integrating SMU is shown in Figure 1. We make the boundary assumption regarding the SoC hardware architecture apart from the on-chip as a trusted domain, and the whole off-chip domain is the untrustworthy region. The self-provided CPU and SPU were highly tested and validated anywhere without potential hardware Trojans inserting in the internal logic; meanwhile, the adversaries cannot tamper the instructions of I-Cache, the dynamic data of D-Cache from on-chip.

According to the different locations where instruction tampering and data tampering are derived from the off-chip interfaces, there are three types of threat model assumptions considered in this paper, which are commonly used approaches to disturb CPU program execution and data processing in reported studies:

- The first situation is that the instructions that were tampered with artificially arise from the program code phase (including software and application) before compiling and linking. For example, the program codes are modified via malicious code injections in C and C++ programmings [19] to cause stack-based and heap-based buffer overflows.
- The second situation is that the program instructions were maliciously modified in external instruction memory (such as flash memory) induced by hardware Trojans. The designers were leaning memory-oriented hardware Trojan insertions to modify or leak memory critical data [20,21]; here, the instruction modifications in memory are manifested as the data bit flips.
- The third situation is that the dynamic data were modified in external main memory by physical tampering attacks (or errors caused by a hardware Trojan). For example, external attacks aim at the vulnerable interface between SoC and external main memory, and their dynamic data exchange suffers from the three physical attacks of bus monitoring, offline analysis, and data tampering [22].

We assume the SoC suffers from external instruction tampering and data tampering attacks. Instruction tampering attacks arise from external instruction memory caused by human or hardware Trojans; therefore, an instruction monitor is implemented in SMU to real-time monitor instruction executions for defending CPU unintended behaviors. In addition, data tampering attacks arise from the external main memory caused by physical detection and tampering, or potential hardware Trojan; therefore, a data monitor is also needed in SMU for achieving high-speed authenticated encryption for data exchanges. In the SoC design, we select the integrity of instruction code as the key parameter of the instruction monitor and the confidentiality and integrity as two key parameters of the data monitor.

## 3. Preliminaries

Our architecture focuses on obtaining the prompt identifications of unintended behaviors via SMU security monitoring at runtime to avoid system malfunction. This section introduces the security strategies related to instruction and data protections in embedded systems, for inspiring the secure SoC architecture design.

### 3.1. Security Strategy of Instruction Execution Monitoring

With the mounting concerns on execution instructions being tampered with in an unauthorized way by adversaries or hardware Trojans, program code integrity and control-flow integrity are the two remarkable techniques for defending the internal intrusions in instruction stream instead of hardware Trojan detection.

#### 3.1.1. Program Code Integrity

The program code integrity strategy checks the integrity of instruction stream to ensure the embedded program does not deviate from the intended and permissible behaviors. In the previous techniques of instruction integrity monitoring, the basic block (BB) signature monitoring scheme according to the program code segments is an effective technology to real-time monitor each instruction’s execution [23]. At the BB granularity, the BB segment of consecutive instructions is defined as when a program starts from the first instruction and ends up with the branch or jump instruction; meanwhile, each BB is assigned to an integrity signature, which is generated on the basis of reference information extraction at the compile phase. During program execution, the integrity signature of each BB is recalculated and compared to the previously on-chip stored one for validating the integrity of BB.

#### 3.1.2. Control-Flow Integrity

Control-flow integrity (CFI) is an effective mechanism for strictly monitoring program execution to see whether it is following the set of possible control-flow transfers, which could be extracted from the statically specified policy of control-flow graph (CFG). Therefore, the CFI checker can detect the unexpected control-flow changes or tampered instructions. The reported protection methods for CFI can be classified into the forward control flow and the backward control flow according to the addressing mode. Forward control flow with an indirect call or jump instruction is often derived from advanced language features, such as virtual functions, function pointers, and callback functions, making it difficult to analyze and implement CFI monitoring in distinguishing different valid targets with a single label. By contrast, backward control flow, such as a return instruction, is relatively easier to protect the return address of a called function and return control back to the calling function in security, such as stack-guard mechanisms of shadow stacks [24] and SafeStacks [10]. Although CFI method can prevent the code-reuse techniques such as return-oriented programming (ROP), the data-oriented programming (DOP) [25] invalidates the CFI monitoring capability via the noncontrol data utilization.

#### 3.1.3. Integrity Label Calculation

The existing solutions for program execution monitoring need to compute integrity labels with the aid of cryptographic hash function and store labels into on-chip memory beforehand. Then, the integrity labels are compared with the recalculated hash values during the program execution. While recalculating the hash values, a suitable cryptographic hash function requires a high-speed hardware to complete the hash calculation of a sequence of instructions. In order to keep up with the CPU execution pipeline as fast as possible, the cryptographic hash function is expected to quickly transform a given sequence of messages into a fixed number of integrity label; moreover, it can keep a low hardware complexity. A reported study [26] proposed a lightweight hash (LHash) function, which employs a Feistel-PG extended sponge structure to improve its diffusion speed in internal permutations. Therefore, we utilize an LHash sequential iteration mechanism in sponge structure to calculate the integrity label of a sequence of instructions during the program execution, while it maintains a low performance overhead.

### 3.2. Security Strategy against Sensitive Data Leakage

As described above, the interface between external main memory and SoC chip is the weakest component to cause dynamic data leakages under the external physical attacks. The adversary can implement bus monitoring and offline analysis to obtain processing data from SoC and, subsequently, can begin to inject tampered data to disrupt program execution. To defend against these threats, a cryptographic algorithm also requires high-speed hardware to keep up with execution pipeline, so that it does not incur a significant performance loss to data processing. At present, the widely adopted strategies for data protection are based on the three cryptographic elements: confidentiality, integrity, and authentication (CIA).

#### 3.2.1. Confidentiality Protection Scheme

Symmetric-key and asymmetric-key are the two frequently used cryptographic algorithms to provide confidentiality assurance for data privacy. Furthermore, considering the sophistication and power of adversary, cryptographic algorithms have to encrypt all the confidential data. As a representation of symmetric-key algorithms, advanced encryption standard (AES) is a block cipher cryptosystem, in which the round function encryption consists of *SubBytes*, *ShiftRows*, *MixColumns* and *AddRoundKey*. Input and output block sizes are fixed to 128 bits, and the different key lengths of 128-bit for 10 rounds, 192-bit for 12 rounds, and 256-bit for 14 rounds are provided according to the required security strength. The AES block-cipher method has a better security feature in confidentiality protection. Furthermore, the RC4 stream cipher proposed in [27] can also provide a good confidentiality protection in data encryption. In addition, the asymmetric-key cryptographic algorithm, such as the Rivest–Shamir–Adleman (RSA), which is a high-quality public key cryptographic algorithm, is suitable for digital signature, key exchange, etc., in a large set of security protocols instead of data encryption [28], because it is so expensive compared to the symmetric cryptography in data-intensive computing tasks.

#### 3.2.2. Integrity Protection Scheme

Data integrity is the assurance of nonalteration. In order to provide integrity authenticity, the cryptographic hash function is required to transform the given amount of data into a digital signature, and any change in input data leads to a large and unpredictable change in digital signature with very high probability. In this way, a receiver can verify the digital signature to guarantee the data have not been modified. For instance, the abovementioned LHash algorithm can be used to provide digital signature for dynamic data. Another famous hash algorithm is named GHash function [29]; by employing the Galois/Counter Mode (GCM), it has advantages in high-speed parallel computations to provide a fast integrity authentication, but its implementation is accompanied by a higher hardware overhead.

#### 3.2.3. Authentication of Digital Signature

The authentication of digital signature requires a good cryptographic hash function to compute data blocks to a suitable size of digest for integrity checker. In embedded systems, the digital signature generated from the extraction of data, address, and timestamp offers a high confidence of preventing an attacker from obtaining the cracked information.

### 3.3. External Tampering Attacks Aiming at Main Memory

**Figure 2:**
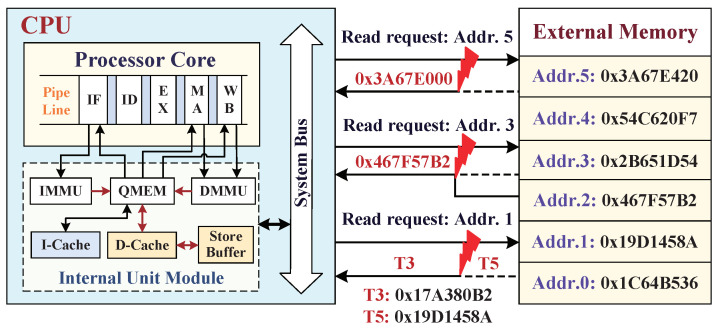
External data tampering attacks: spoofing attack, relocation attack, and replay attack.

In order to better understand the embedded system external data tampering attacks, the processor architecture suffers data tampering attacks from external main memory, as shown in Figure 2. Data tampering attacks can be classified into three types of attacks: the spoofing attack, corresponding to read-load request *address 5*, which exploits a partially modified data to camouflage such as a legitimate data to replace the correct data block, causing the processor to malfunction; the relocation attack, which occurs at read-load request *address 3* and utilizes the data block in *address 2* to swap the returned data block from *address 3*; the replay attack, which happens at read request *address 1* and exploits a previously stored data block at time *T3* to replay the data block at read-request time *T5*. According to their different attack capabilities, the relocation attack is more easily tricks the processor into accessing unauthorized data compared to the spoofing attack. Since these data blocks in external memory are encrypted with the same scheme, an attacker could tamper the processor behaviors by swapping some encrypted values. What is worse, the replay attack at the different time can easily overcome the protections against the relocation attack to modify the processor behaviors. Therefore, the uniqueness of encrypted data in time and space is applied in the data monitor to resist the above three types of data tampering attacks.

## 4. Instruction Monitor against Instruction Tampering Attacks

In this section, we consider the characteristics of instruction executions to efficiently implement the instruction monitor. This section contains four main components: the efficient partition of basic blocks (BBs) at appropriate granularity; the reference information extraction of BBs for real-time integrity checking; the hardware implementation of instruction monitor; and the performance optimizations of a monitoring mechanism.

### 4.1. The Efficient Partition of Basic Blocks

The previous report in [30] indicates that the performance overhead of a hardware monitor is relevant to integrity checking speed; further, the speed of validation depends on BB granularity. When the BB partition of program code is at a coarse granularity for reducing the number of program integrity verification, this BB integrity monitoring granularity for reducing performance overhead may be overlarge, because the large number of transfer instructions require frequent jumps and function calls. In addition, BB contains a variety of possible program execution orders, which make it difficult to analyze and extract the unique and effective BB reference information, even causing the monitor to not be sensitive to instruction damage issues such as injection, deletion, and tampering. When the BB partitions are too small, even with each instruction as a BB, this fine-grained monitoring method causes the instruction monitor calculation for BB integrity signature to not be able to keep up with CPU execution pipeline. Hence, a suitable BB granularity contributes to achieving high security and low overhead.

During processor executing program instructions, the instruction counter is regularly incremented by one; meanwhile, the instruction is read from next target address to processor until the jump instruction is encountered. The transfer-type instructions cause the program execution discontinuity and have many possible execution directions. Hence, we plan to partition the program instruction stream with the BBs strictly according to the branch and jump instruction characteristics. Each BB is composed of a group of successive instructions so that BB is executed sequentially. We defined that each program BB starts from the first instruction and ends up with the branch or jump instruction. This partition strategy might appear in the overlaps of BBs, which aid in reducing the number of searching labels in BBs reference information table ($TAB_{BB}$) at the same storage spaces compared to the other nonoverlapping partition strategy.

**Figure 3:**
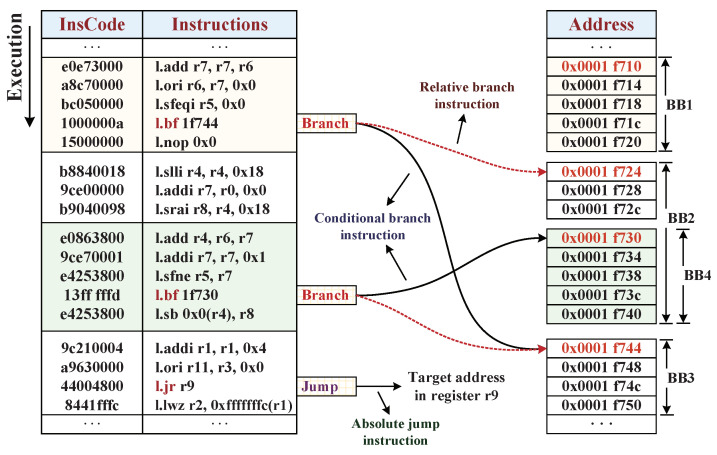
An example of the benchmark OpenECC instruction segmentation for illustrating the proposed partition strategy of BBs.

As shown in Figure 3, a segmentation of instruction code from the benchmark of OpenECC was selected to illustrate the partition strategy of BBs. Firstly, the instruction stream can be partitioned into BB1, BB2, and BB3 fluently according to the boundary of transfer-type instructions (branch and jump). Secondly, the instruction transfer target address of each BB can be deduced according to transfer-type instruction. For example, the instruction *l.bf* is a conditional branch instruction, and two possible legal branch addresses can be inferred from the analysis of the instruction code. The absolute jump instruction *l.jr* jumps to the target address corresponding to the value of r9 register, which is usually the returned address of the superior function. Although the value of r9 register cannot be extracted in offline analysis, a new BB can be generated by processing the function entry address and traversing its target jump address. Considering the target addresses of two *l.bf* conditional branch instructions, BB3 and BB4 can be generated, where BB3 is an overlap with previous BB3 and the BB4 is inside the BB2 from a new start address.

It is noteworthy that in our partitioning strategy of program code with BBs, we considered the delay slot mechanism to reduce CPU performance loss. The branch delay slot is the wasted clock spaces following the conditional branch and jump instructions. In the processor five-stage pipeline, it requires three clock cycles to complete a branch instruction execution, which include instruction fetch, instruction decode, and instruction execution, and then jumps to another target address and causes the pipeline discontinuity. To improve the execution efficiency in clock cycles, the delay slot instruction is also partitioned into each BB for filling the pipeline clock gap, where it follows the branch or jump instruction as the end boundary of each BB. This BB partitioning strategy minimizes performance loss.

### 4.2. Reference Information Extraction for Integrity Checking

The purpose of the partitioning program code with BBs in this work is achieving modular security checks for the instruction monitor with minimal performance loss and high security. In the design of instruction monitor, the reference information ($INF_{ref}$) of divisiory BBs should be predefined to determine each BB integrity monitoring parameter. For satisfying the security monitoring requirements against the various forms of instruction tampering attacks and achieving a quick integrity verification, the selected security parameter requires it to meet these three conditions: (1) it must be sensitive to any damage issue, seeing as the injection, deletion, or tampering of instruction causes the security parameter to change; (2) it is easy to extract from each BB; and (3) considering the limited hardware resource of embedded system, it should be minimized while ensuring adequate security sensitivity.

After the above comprehensive consideration, we plan to extract the effective start address ($ADD_{start}$) of each BB, the instruction code (InsCode), and the BB digest generated by using the LHash function ($DIG_{lhash}$) to constitute the expected 32 bit integrity monitoring $INF_{ref}$, organically. The adopted OpenRISC processor OR1200 has 32 bits instruction code and target address, whose instruction and address are aligned to 4 bytes. Due to the lower 2 bits of a 32 bit instruction address in program counter (PC) being fixed to 2’b00 (addressing RAM by word), the available value as the effective start address of each BB is PC[31:2]. In general, the width of a 32 bit address can provide 4 GB address space, where the PC[31:2] value of BB start address leads to a large on-chip storage consumption constituting the integrity reference information of $INF_{ref}$. Therefore, we selected the lower 16 bits effective values from PC, that is, PC[17:2], as the start address value of BB in $INF_{ref}$, which can provide the applications up to 256 KB address space. Furthermore, the size of address space can be extended by selecting more effective bits from PC[31:2] according to real application requirements, and its storage resource overhead also increases.

In this work, we employed the abovementioned LHash algorithm to generate each BB digest $DIG_{lhash}$ for performing InsCodes integrity verification in instruction monitor. We selected 32 bit InsCodes and start address (for identification) of each BB as the input message blocks into LHash engine’s sponge structure. After the segment of consecutive BB InsCode being calculated by the LHash algorithm, a high-security 96 bits BB LHash digest was obtained. Considering that the obtained 96 bits LHash digest causes a large on-chip storage resource overhead constituting $INF_{ref}$ table in monitor memory, we selected a 16 bits available value from the 96 bits LHash digest according to the bit-selected numbers from a offline random number generator (RNG), for creating a 16 bits golden LHash digest $DIG_{lhash}$ to avoid an attacker forging the valid digest value. Finally, the 32 bits $INF_{ref}$ are composed of the 16 bits $ADD_{start}$[17:2] and the 16 bits golden $DIG_{lhash}$, where the $INF_{ref}$[31:16] is assigned with $ADD_{start}$[17:2], and the $INF_{ref}$[15:0] is assigned with $DIG_{lhash}$.

**Figure 4:**
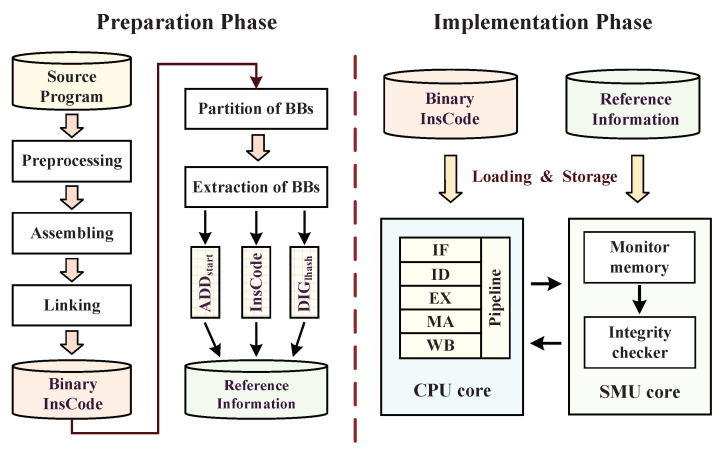
The static extraction preparation phase of reference information and the implementation phase for real-time monitoring.

The offline extraction preparation phase of reference information and the implementation phase of real-time monitoring are shown in Figure 4. In which, the preparation phase mainly consists of the compile and link processes, the partition of BBs, the security parameters extraction, and the constitution of $INF_{ref}$. In preparation, the GNU tool or32-elf-objdump was utilized to disassemble the binary InsCode, we employed the regular expression to search all the function entries, jump instructions, and destination addresses. The implementation phase depicts $INF_{ref}$ memory storage in the instruction monitor while loading program binary InsCodes for execution.

### 4.3. Hardware Implementation of Instruction Monitor

**Figure 5:**
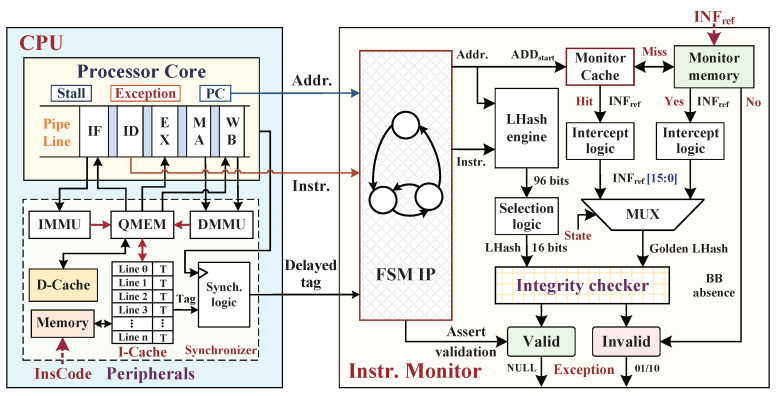
The hardware implementation details of the instruction monitor.

After completing the offline preparation works of BB partition and $INF_{ref}$ extraction from InsCodes, the hardware implementation design of instruction monitor should provide a high-efficiency violation detection in the instruction stream integrity at BB granularity. Figure 5 shows the hardware architecture details of the instruction monitor. The instruction monitor checks the integrity digests of BBs according to the execution order of source program. The hardware-assisted instruction monitor takes InsCodes and $ADD_{start}$ as the input signals, where InsCodes and $ADD_{start}$ are exported from the instruction decoding (ID) stage of execution pipeline and PC, respectively. We provide FSM IP to keep track of transfer-type instructions (branch and jump) through control-state transitions, which can identify the BB boundary of start address (also being the target address of previous jump instruction) and end address with delay slot instruction after the branch/jump operation. After FSM, the instruction streams of each BB are continuously pumped into the LHash engine when $ADD_{start}$ is detected, and then the LHash engine recalculates the 96 bits LHash digest of each BB while the CPU executes the sequence of InsCodes. Finally, the 16 bits LHash digest ($DIG_{cal}$) can be generated by stored specific bit-selected numbers according to RNG. Meanwhile, in another path, the configured monitor cache (M-Cache), which associatively maps with monitor memory (for storing $INF_{ref}$), searches the cache lines according to the received start address ($ADD_{start}$) of each BB. If the M-Cache hits, the corresponding M-Cache line of $INF_{ref}$ block is input to an intercept logic for obtaining the $INF_{ref}$[15:0] as golden LHash ($DIG_{lhash}$); if M-Cache misses, the instruction monitor starts to search the $ADD_{start}$ in monitor memory. If it succeeds, two inputs multiplexer (MUX) controlled by the states of hit/miss receive the $INF_{ref}$[15:0] after the intercept logic; if it fails to search, the monitor asserts an invalid signal of BB absence to the processor. When M-Cache hits or memory hits, the recalculated $DIG_{cal}$ is compared with the stored $DIG_{lhash}$ in integrity checker. The instruction monitor asserts valid BB when their compared result is equal. Otherwise, the instruction monitor asserts the BB as an invalid status, where we preset the LHash value error with invalid status “01”, and the start address error with an invalid status “10” (BB absence).

The invalid signal is sent into the exception module when instruction monitor detects a violation of BB integrity, which is nonmaskable to trigger the fast-response mechanisms inside the processor. In general, when the instruction fetching (IF) stage in pipeline fails to read instructions from I-Cache, it needs to fetch instructions from instruction memory, and the CPU sends out a CPU_STALL signal to suspend the execution pipeline due to the absence of execution instruction. In the instruction monitor, the integrity checking of each BB waits for all of the instructions in the current BB being executed. Therefore, while checking the BB integrity, instruction monitor still asserts the CPU_STALL signal until successful integrity checking.

### 4.4. Performance Optimizations of Monitoring Mechanism

An important consideration of the instruction monitor is how to reduce its performance overhead during BB integrity checking during the instruction execution. For each BB, the first instruction is the entry of BB, and the end of BB is the delay slot instruction, which the upper instruction leads the instruction stream to branch or jump to a new start address of the other BB. Due to LHash calculation and $INF_{ref}$ searching needing to consume some clock cycles, it is possible that all the instruction executions of a BB are completed, and the comparative result of the BB integrity checker is not yet asserted, which affects processor performance. We configured an M-Cache and optimized the I-Cache to improve the searching efficiency of $DIG_{lhash}$ in the instruction monitor.

#### 4.4.1. M-Cache Searching Method

**Figure 6:**
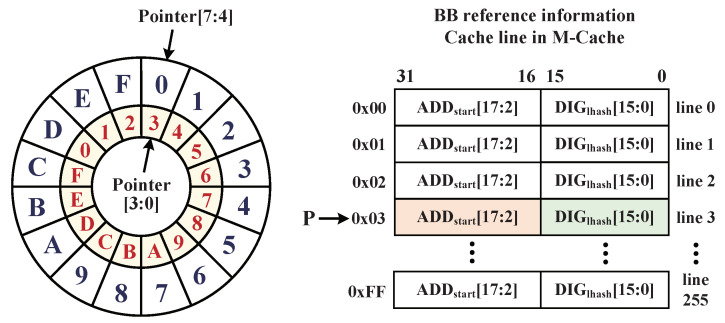
The M-Cache searching method and internal structure of BB reference information table.

When the instructions of each BB are executed, the $ADD_{start}$ of BB is first sent to M-Cache for searching cache lines, and the corresponding $INF_{ref}$ can be obtained directly from cache lines if the M-Cache hits. This method can avoid the frequent access of monitor memory. For improving the hit rate of M-Cache, a depth of 256 cache lines is configured for M-Cache to the buffer partial $INF_{ref}$ blocks of BBs from monitor memory. The M-Cache searching method and the internal structure of BB $INF_{ref}$ table are shown in Figure 6. The content-searching method of M-Cache pointer is described as a double ring buffer that is constructed with one 8 bits register. Meanwhile, the storage parts of $ADD_{start}$ and searching circuit are fully interconnected, so that the hit status of M-Cache can be acquired within two clock cycles. In the M-Cache, the pointer[7:0] searches the M-Cache according to the BB start address segment of $ADD_{start}$[17:2].

#### 4.4.2. I-Cache Optimized Approach

To further decrease the performance overhead caused by LHash recalculation and searching the $INF_{ref}$ table, the I-Cache can be optimized to reduce the number of times on BBs integrity checking. We utilize the locality principle of I-Cache mapping InsCode memory to tag the instructions of BB, with those that were cached in I-Cache and were validated for integrity during other BB executions. An I-Cache line has four instruction words, and when the four instructions at same cache line are read for execution, the Tag bit in the cache line turns from “0” to “1” to indicate the instructions in the current cache line being verified for integrity. From the partition principle of BBs, a BB contains at least three instructions and occupies one or two cache lines, the long BB occupies several cache lines. Hence, I-Cache outputs the Tag signal of security when all the instructions of the BB are cached in I-Cache, and the Tags of cache lines they occupied are all signed with “1” ( for logic AND). Then, the delayed Tag from a synchronizer input into the above FSM controller, and instruction monitor directly asserts the validation of the processor. This optimized approach plays an important effort to reduce BB integrity checks on the situation that the BB overlaps with the other BBs.

**Figure 7:**
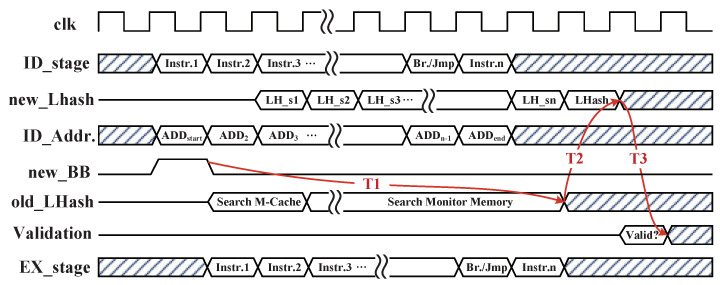
The timing diagram of a BB execution with integrity validation at the worst situation.

In the abovementioned optimization, the configured M-Cache and I-Cache improve the searching efficiency of $INF_{ref}$ block. It is noteworthy that the worst situation occurs when M-Cache and I-Cache are both failed to complete integrity verification for the current BB. Therefore, the instruction monitor needs to search the whole $INF_{ref}$ blocks table in monitor memory. Figure 7 depicts the timing diagram of one BB execution with integrity validation at the worst situation. The period of T1 represents the total time consumption spent searching the $INF_{ref}$ block in both M-Cache and monitor memory from a new BB being detected, and its search result can be obtained with a high probability before the recalculated result of LHash engine. Period T2 represents the time consumption of golden LHash $DIG_{lhash}$ being obtained and waiting for verification. Period T3 indicates that the integrity checker completes the comparison and outputs the validation status within one clock cycle. Since the searching process of $INF_{ref}$ according to the $ADD_{start}$ of each BB in M-Cache and monitor memory is simultaneous with instruction executions, it can minimize the performance overhead of BB integrity checking; thus, the time consumption on searching $INF_{ref}$ in M-Cache and monitor memory are both acceptable for integrity validation.

## 5. Data Monitor against External Physical Attacks

In this section, we describe the hardware implementation details of data monitor on preventing dynamic data leakage and data tampering from external physical attacks; meanwhile, we expatiate the dynamic monitoring mechanism of data monitor for achieving a superiority of low performance overhead.

### 5.1. Hardware Architecture Implementation of Data Monitor

**Figure 8:**
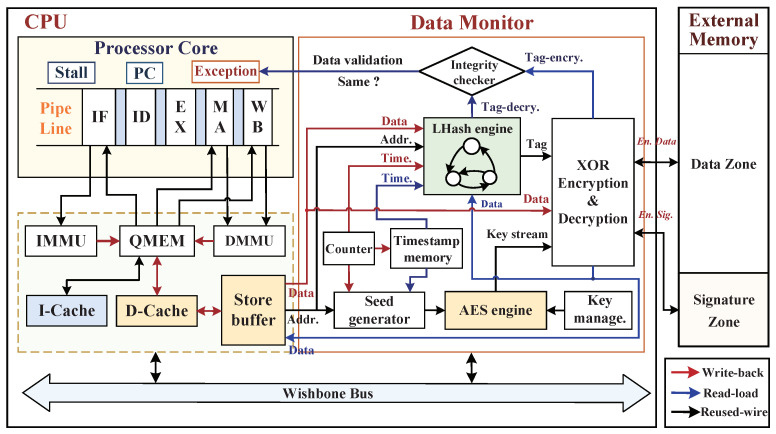
The hardware implementation of the data monitor connected with the CPU.

Our proposed data monitor is a part of the SMU that connects with the CPU core via D-Cache and store buffer modules, and the overall hardware implementation architecture is shown in Figure 8. In which, the hardware-realized data monitor was applied between the store-buffer and external main memory for providing dynamic data confidentiality and integrity protections during the program execution of processor.

The hardware-implemented data monitor integrates the AES engine, LHash engine, seed generator, counter, key management unit, integrity checker, etc. In which, the AES engine is a symmetric-key block cipher cryptosystem that supports input and output data lengths both at 128 bits, and we deploy the length of key as 128 bits (with 10 rounds). Its 1–9 round encryptions are duplicated in the four transformations of *SubBytes*, *ShiftRows*, *MixColumns*, and *AddRoundKey*, and round 10 without *MixColumns* transformation. The AES engine can provide a good confidentiality protection for the dynamic data exchanges to external memory against the bus monitoring and offline analysis attacks. For responding effectively to the physical tampering attack, LHash engine is adopted to provide integrity protection and Tag integrity verification. In addition, an integrity checker is utilized to check the validation of the integrity tag and send valid or invalid signals to the CPU exception interrupt module. Timestamps are generated by increasing the counter with increments of one, then the count values are stored into on-chip timestamp memory to ensure the time uniqueness of AES key stream.

In the procedure of CPU loading/storing data blocks, the CPU core first sends the request effective address for loading or storing data, and then data memory management unit (DMMU) identifies the address offset to determine a physical address and sends it to QMEM. QMEM judges the physical address to see whether it is within its address space range. If it is, the CPU reads or writes the specific address directly; if it is not, QMEM sends the request address to D-Cache, and then the D-Cache searches the physical address to see whether it has been cached. If the D-Cache hits, the CPU reads/writes data depending on the appointed physical address; if the D-Cache misses, then CPU reads/writes data via accessing the external main memory. Between the D-Cache and data monitor, the store-buffer and WB_BIU are configured as shown in Figure 8, where the WB_BIU module is hidden for the sake of brevity. Due to the main memory being located in a vulnerable domain and facing a risk of being attacked maliciously, the data monitor is activated only when the D-Cache addressing misses (on Write-Back method) achieving a good trade-off between security and performance overhead. In the proposed data monitor, we distinguish the data write-back and read-load procedures with red and blue arrows, respectively, and the reused signals with black arrows are employed in both write and read procedures.

### 5.2. Data Write-Back Procedure of Memory Access

The hardware-implemented data monitor is an efficient technique to complete high-speed runtime data encryption operations with a reasonable hardware overhead. However, executing the real-time encryption and decryption operations for all requires the write-back data to be impracticable in executing the intensive data processing tasks, because the excessive encryption protections cause the processor to have a large performance overhead. Combined with the superiority of the D-Cache on the locality principle of mapping a memory, we adopted the write-back method which does not write data to external main memory synchronously when the CPU writes to the D-Cache (D-Cache hits). Here, the D-Cache is inconsistent with the main memory on data blocks, so that external attacks do not cause dynamic data leakage or function failure before the main memory is overwritten. If the D-Cache misses, the CPU directly accesses external main memory to write back according to the access address, while the D-Cache begins to stay consistent with the main memory. In their data synchronization, many dynamic data blocks in the D-Cache require encryption protections before storing to the external main memory at one time. The data monitor is activated when the D-Cache addressing misses, and the “dormancy” mechanism on the data encryption operation plays an important role in decreasing the number of times of accessing the external main memory and performance overhead.

#### 5.2.1. AES Ciphertext Generation

**Figure 9:**
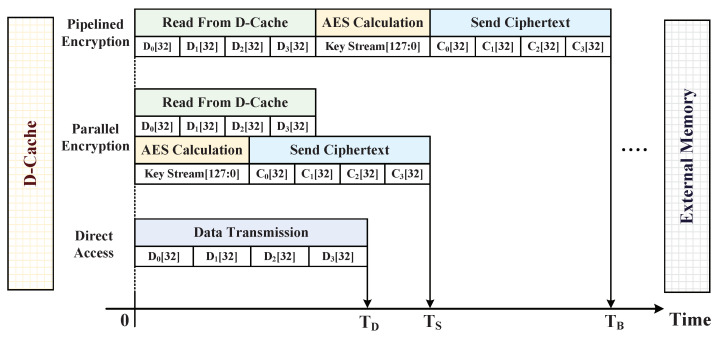
Time consumption comparison of the three different methods for completing a write-back operation from D-Cache.

The CPU is a Harvard RISC processor with 32 bits instruction bus and data bus, and the D-Cache line size is set as 16 bytes (128 bits). Generally, the data block encryption in AES engine requires the acquisition of a complete 128 bits data block as being input; therefore, the data bus needs to access D-Cache four times to obtain a complete 128 bits data block. Then, the AES engine works to encrypt and output the ciphertext, but this pipelined encryption method significantly reduces 128-bit data block encryption efficiency. In order to improve encryption efficiency for AES engine, we adopted a parallel encryption method instead of the pipelined encryption method to encrypt the request physical addresses, which correspond to the write-back data blocks. The time consumption comparison of the three different methods for completing a write-back operation from D-Cache is shown in Figure 9. In the parallel encryption method, the AES engine calculating 128 bits key stream is parallel to the data block transmission process. During the transmission process, the received 32 bits data sub-blocks begin to generate ciphertext sub-blocks by performing the XOR operations with the AES key stream. Therefore, the parallel encryption method can save the time $T_{B}$-$T_{S}$ compared to the pipelined encryption method, and the encrypted process of data block can be represented as follows.

(1) $$C=P⊕AES_{KEY}\left(Seed\right)$$

where *C* and *P* denote the ciphertext and the plaintext of the data block, respectively. $AES_{KEY}\left(Seed\right)$ is expressed as the key stream generated from the AES engine.

Using the key management unit to provide a 128-bit initial key for the AES engine’s key expansion and 10-round operations. To improve the confidentiality of data blocks, their physical address and timestamp are inputted into the seed generator for ensuring the uniqueness of the AES key stream on space and time. The internal function structure of seed generator is the bit-wise Exclusive-OR operation, in which the AES seed and timestamp have the same bit widths with the 32 bits physical address.

#### 5.2.2. LHash Digital Signature Generation

While protecting the confidentiality of the key stream from the AES engine, the sponge construction of the LHash engine absorbs the physical address, timestamp, and data block sequentially, and the absorb procedure can be controlled by an built-in FSM controller. The LHash engine first absorbs the physical address of the 32 bit data sub-block ($D_{0}$); the second step absorbs the timestamp corresponding to $D_{0}$; and the third step absorbs the four 32 bit data sub-blocks ($D_{0}$-$D_{3}$) orderly. Finally, the LHash engine outputs a 32 bits hash integrity Tag. Furthermore, the encrypted digital signature is created by performing the LHash Tag XORed with the AES key stream. It is noteworthy that our protective granularity of data block is at 128 bits, which is exactly same with the D-Cache line size, and the latter consists of four 32 bit continuous data sub-blocks. Thus, we set the line size as a whole unit to participate in AES encryption and LHash calculation for avoiding the errors caused by sub-blocks order in the decryption process.

#### 5.2.3. Data Write-Back Procedure with D-Cache

**Figure 10:**
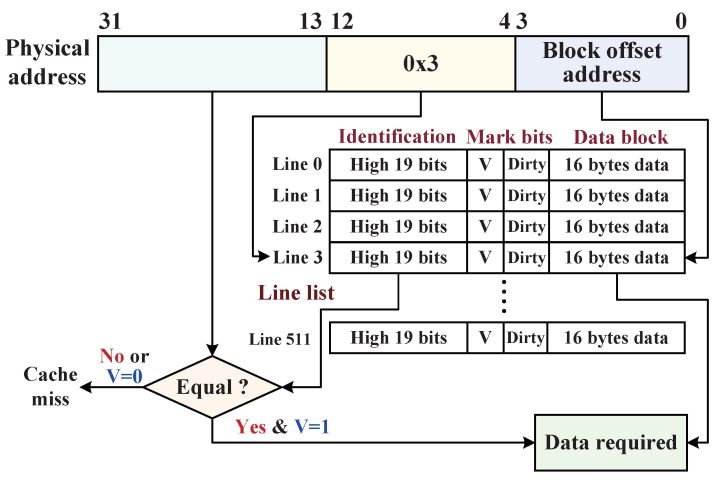
The implementation mechanism of the D-Cache with the size of 8-KB.

In addition to improving the data processing efficiency, the D-Cache principle of locality dynamically activates the data monitor to encrypt and decrypt data blocks. Figure 10 illustrates the internal implementation mechanism of the D-Cache. When CPU needs to write-back a 32 bit data sub-block to the external main memory, a 32 bit physical address ($ADD_{phy}$) corresponding to the data sub-block is sent to the D-Cache through the address bus. $ADD_{phy}$ consists of three parts, and its high 19 bits $ADD_{phy}$[31:13] are utilized to be compare to the high 19 bits identification tag of the indexed cache line appointed by the $ADD_{phy}$[12:4] (cache-line depth with 512). If their values after a comparison are equal, while the *Validity* (*V*) mark bit in appointed cache line is “1”, which indicates the D-Cache hit so that the physical address can accurately find its target address according to the block offset address of $ADD_{phy}$[3:0], then the original data sub-block of target address will be overwritten by the write-back sub-block. Once a target address completes the data overwriting (where main memory not updated), the *Dirty* bit in the cache-line turns from “0” to “1”. Otherwise, the D-Cache searches miss when their values are unequal, which indicates the write-back target address was not cached in D-Cache or the physical address is appointed to an invalid cache line (V = “0”), in which the data block of cache-line is invalid. Afterward, the D-Cache caches the corresponding cache line from the external memory according to the physical address via the direct mapping method, and then, the CPU completes the overwriting operation, and the line *Dirty* bit is marked with “1”. Finally, the D-Cache synchronizes the data blocks (*Dirty* =“1”) to the main memory with encryption.

When the D-Cache addressing misses, the CPU prepares to write back data blocks to external main memory, and data monitor is activated immediately. Algorithm 1 describes the write-back procedure of a 128-bit data block with authenticated encryption protection. The hardware implementation of this procedure is shown in Figure 8. Ultimately, the ciphertext block (4×32 bits sub-blocks) and its relevant encrypted digital signature are stored into the data zone and signature zone of the main memory, respectively.
**Algorithm 1** Write-back operation of 128-bit data block stored into external main memory**Inputs:***Data*, *Address***Outputs:***Timestamps*, *Ciphertext*, *Signature*1:*Data*← set of *data*${}_{i}$ sub-blocks to write back, 0≤i≤3.2:*Address*← set of memory physical *address*${}_{i}$, 1≤i≤3.3:**D-Cache Misses**, the data monitor is activated;4:**begin**: Data monitor detects a write-back signal, counter generates a timestamp (Ts), Ts + 1, buffered in Ts memory;5:**input**: four physical address blocks, four *data*${}_{i}$ orderly;6:**for all***Address*${}_{i}$ (i = 0; i++; i ≤ 3) **do**7:      $Seed_{i}$ = $address_{i}$ XOR *timestamp*;8:      *Key_stream* = $AES_{KEY}\left(Seed\right)$;9:      *Ciphertext* = Data XOR *Key_stream*;10:**end for**11:**for all***data*${}_{i}$ (i = 0; i++; i ≤ 3) **do**12:   $LHash_{tag}$ = $f_{LHash}$($Address_{0}$, timestamp, $data_{i}$);13:   Signature = $LHash_{tag}$ XOR *Key_stream*14:**end for**15:Data zone ⇐Ciphertext, Signature zone ⇐Signature;

### 5.3. Data Read-Load Procedure from External Memory

When CPU requests to read-load a 32 bit data sub-block from the external main memory, the specific physical address is sent to the D-Cache for searching. If the identification value of $ADD_{phy}$ [31:13] is same with the high 19 bits identification tag of cache line (appointed by the $ADD_{phy}$ [12:4]), while the line mark bit of *Validity* is “1”, this indicates the D-Cache hits and then it cached data sub-block corresponding to the physical address sent to the CPU directly. Otherwise, if the D-Cache misses, the D-Cache needs to read four data sub-blocks (one cache line) from external main memory, and then four physical addresses are orderly sent into the data monitor and the external memory. In the data monitor, the timestamp memory pops the timestamp of the address to create the AES key stream and LHash integrity tag. Meantime, the read-load signal synchronously feeds back the data ciphertext block and its corresponding encrypted signature in external memory, and then, the ciphertext block and signature are read into the data monitor for decryption and integrity checking. The decryption process of ciphertext block is represented as follows.

(2) $$P=C⊕AES_{KEY}\left(Seed\right)$$

where *P* and *C* denote the plaintext and the ciphertext of 128-bit data block, respectively. In addition, the integrity tag in encryption period (*Tag-encry.*) can be obtained by performing the digital signature XORed with AES key stream, and the LHash engine calculates data integrity tag in the decryption period (*Tag-decry.*). Finally, the tag of *Tag-decry.* in decryption period is compared with the *Tag-encry.* of the encryption period in integrity checker. Once their comparison values have violated the integrity checking, an exception signal is sent to the exception unit of CPU. The read-load procedure of a ciphertext block with the integrity verification from the external memory is described in Algorithm 2, and its hardware implementation is shown in Figure 8. The dynamic data protection technique is complementary to the instruction monitoring technique against the dynamic data leakage and data tampering.
**Algorithm 2** Data-load operation of 128-bit ciphertext block from external main memory**Inputs:***Address*, *Ciphertext*, *Signature***Outputs:***Data*, *Exception*1:*Ciphertext*← set of the *ciphertext*${}_{i}$ sub-blocks, 0≤i≤3.2:*Address*← set of memory physical *address*${}_{i}$, 1≤i≤3.3:**D-Cache misses**, the data monitor is activated;4:**begin**: Data monitor detects read-load signal and physical address, timestamp (Ts) memory pops out the correlative Ts into seed generator and LHash engine;5:**input**: four address blocks, four *ciphertext*${}_{i}$, Signature;6:**for all***Address*${}_{i}$ (i = 0; i++; i ≤ 3) **do**7:      $Seed_{i}$ = {$address_{i}$ XOR *timestamp*};8:      *Key_stream* = $AES_{KEY}\left(Seed\right)$;9:      *DATA* = *Ciphertext* XOR *Key_stream*;10:**end for**11:**for all***data*${}_{i}$ (i = 0; i++; i ≤ 3) **do**12:   *Tag-decry.* = $f_{LHash}$($Address_{0}$, timestamp, $data_{i}$);13:   *Tag-encry.* = *Signature* XOR *Key_stream*14:**end for**15:**if***Tag-decry.* = *Tag-encry.* **then**16:      *Exception* = null (“00”)   /* integrity valid */17:**else***Exception* = assertion (“11”);   /* integrity invalid */

## 6. Experiments and Results

This section presents the experiments and results of SoC to expatiate features of instruction monitor and data monitor in SMU on performance overhead and security capability.

### 6.1. Experimental Setup

We implement the proposed SMU into the OR1200 embedded system for ensuring the instruction execution and data processing security, and the basic frequency of this scalar RISC processor core is set as @100 MHz, and the internal clock cycles satisfy the synchronization with the extended SMU. The hardware configurations of I-Cache and D-Cache support the different sizes of 2, 4, 8, and 16 KB. We first configured the processor with a typical depth size of 8-KB I-Cache and 8-KB D-Cache, in which the internal structures consisted of the 512 cache line blocks. We developed the secure embedded system with SMU in Verilog HDL and performed the logic synthesis and implementation in Xilinx ISE Design Suite 14.7. This SoC hardware architecture with SMU was evaluated on a Xilinx Virtex-5 FPGA platform, and GNU Cross Compilation Toolchain or32-elf-gcc matching with OR1200 instruction set architecture (ISA) was utilized to generate InsCodes. Moreover, we configured some necessary controllers for the off-chip peripherals, such as the DDR2 SDRAM, parallel flash, serial ports, and Ethernet. In the operational system initialization stage (Boot Process), the SoC bitstream first is programmed from the flash memory onto FPGA at power-up; then, the bootloader (U-Boot) boots the Linux kernel to mount the root file system for execution. We adopted the direct mapped cache with external main memory, while we defaulted that the initial data stored in main memory is secure and SMU encrypts the dynamic data during the whole application life cycle.

### 6.2. Performance Overhead Evaluation

While the CPU executes the instruction codes, the integrated SMU inevitably causes the performance overhead in the embedded system. In the proposed SoC hardware architecture, we made some efforts to reduce the performance overhead aiming at instruction monitor and data monitor, such as the optimizations with I-Cache, M-Cache, and D-Cache. In the experiments of the performance evaluations, we selected ten various scales embedded in the benchmarking programs from Mibench suite [31] to execute real applications. The MiBench group is developed based on the EEMBC-CoreMark, which consists of 35 applications and spreads across the six classes embedded application scenarios. They are popular performance benchmarks in academia, industry, and the military. The selected ten benchmarks first are preprocessed under offline analysis and static extraction via running Perl scripts to generate the $INF_{ref}$ of BBs; then, the benchmarks are compiled by GNU Cross Toolchain or32-elf-gcc and downloaded into FPGA for program execution, respectively. Furthermore, the numbers of total instructions and all BBs of each benchmark are counted, and the $INF_{ref}$ table required on-chip storage space is also calculated. Considering the hit rates of I-Cache and D-Cache can influence the performance overhead, we used the or1ksim [32] simulation software to record the hit rates of the I-Cache and D-Cache, respectively. Hence, the system average performance overheads of the different benchmarks can be calculated according to the indicator of cycles per instruction (CPI) on the SoC with and without integrating the SMU.

#### 6.2.1. SMU Performance Overhead

The overall performance overhead of the SoC configured with the SMU is shown in Table 1. These experimental data present that the benchmark of OpenECC has the largest number of instructions and BBs, and its $INF_{ref}$ blocks require a maximum on-chip storage space of 26.30 KB in monitor memory. We also found that the average hit rates of I-Cache and D-Cache configured with 8-KB both exceeded 98%, and this superiority means that their high-hit rates can effectively keep a low performance overhead with the SoC integrating the SMU. The benchmark of AES has the highest D-Cache write-hit rate and read-hit rate, with both being beyond 99.5%. Its arithmetic characteristics determine a higher proportion in the data write-back and read-load operations, and the data monitor causes the performance overhead to be a little higher. The indicator CPI tends to increase with the number of benchmark instructions, which means the memory access instruction occupies a large proportion in InsCode. For example, running the benchmarks of OpenECC, basicmath, and patricia requires a large number of external main memory access, and the data monitor is activated multiple times in response, and their CPI values are higher than the other benchmarks. Finally, the experimental data indicate that the average performance overhead of SoC is 2.18%, ranging from 0.54% (quicksort) to 4.09% (OpenECC).

#### 6.2.2. Different Depths of M-Cache

To further explore the efforts of M-Cache in reducing the performance overhead caused by SMU, we made experimental statistics about the indicator CPI under different depths of M-Cache. In order to find the suitable depth which can reach a good tradeoff between storage space and performance overhead, M-Cache depths are configured with the several cases of no M-Cache, 16, 32, 64, 128, and 256. The performance overheads of processor configured with the different depths of M-Cache are presented in Table 2. Due to the raising in depth of M-Cache, CPIs continuously reduce, while the M-Cache has higher hit rates, and its reduction trend slows down when the hit rate reaches a saturation. Therefore, we integrated the M-Cache with depth(256) into the SMU for achieving a good performance overhead of 2.18%.

#### 6.2.3. Different Sizes of D-Cache

**Figure 11:**
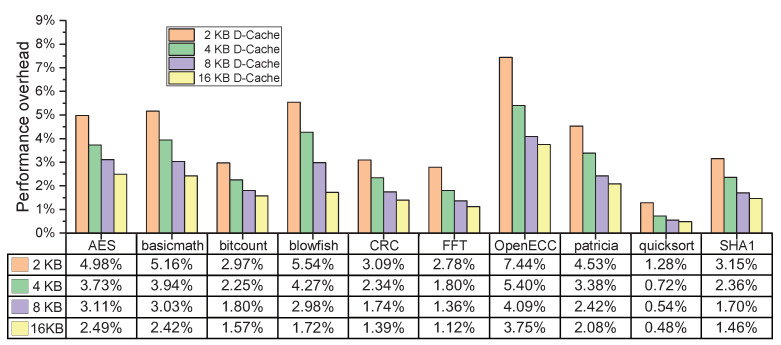
The performance overheads of the selected benchmarks under different sizes of D-Cache.

To explore the influences of the D-Cache about the hit rate to system performance overhead, we continue the evaluation experiments by keeping the I-Cache 8-KB unchanged, and the size of D-Cache is reconfigured as 2, 4, and 16 KB, respectively. Figure 11 shows the performance overheads of ten selected benchmarks under the different sizes of D-Cache. Due to the cache enlargement in the addressing space, the performance overhead decreases with the increase in D-Cache hit rate. At the largest configuration of the 16-KB cache, the performance overhead of SoC (including SMU) has a significant reduction and ranges from 0.48% to 3.75%. The mechanism of this trend is that while the D-Cache hit rate increases, and the number of times of data monitor in SMU being activated decreases, because the authenticated encryption operations incur the additional clock cycles. Moreover, the size of the 8-KB D-Cache can better reduce the performance overhead incurred by SMU, which is a suitable size for SoC in the real applications at reasonable resource consumption and hardware complexity.

### 6.3. Security Capability Evaluation

In order to confirm the effectiveness of SMU on instruction and data monitoring, the OpenRISC debugging system OR1K was established to observe the program execution flow, special register modification, memory access, exception, or interruption record. Hence, we performed the instruction tampering evaluations in the offline preparation phase of binary instruction codes. Taking part of the instructions of the benchmark OpenECC (as shown in Figure 3) to be evaluated, the nontransfer instruction *l.nop 0x0* is artificially tampered with as *l.nop 0x1*, and the branch transfer instruction *l.bf 1f730* is modified to the different branch address of *l.bf 1f734*. Meanwhile, we performed the data tampering experiments at runtime by injecting modified data into external data bus. While the CPU executes the benchmarks, the condition of D-Cache missing was created artificially, and the three types data tampering attacks were implemented to modify the ciphertext sub-blocks on the data bus from external memory, respectively. The debugging system plays an important role in directly communicating with the CPU and the Wishbone bus, for starting and breaking the executions of benchmarks. Table 3 presents the security capability tests of the SoC with SMU under different data tampering attacks. Their output exception binary results are analyzed, and the corresponding log files are displayed in the upper machine.

According to the error_log files, the integrity verification for binary instruction codes at granularity of BBs can recognize any instruction tampering behaviors in transfer and nontransfer instructions. For the nontransfer instruction, the instruction monitor only asserts the LHash error (“01”), which means BB integrity checking failed, and then the error_log reports the corresponding BB number and correct LHash value. There are two invalid statuses for the transfer instruction, and the LHash error (“01”) is first reported when current BB integrity checking failed, and then another BB absence (“10”) is reported when BB start address (target address of branch or jump) searching miss. In the experiments, data monitor only asserts the integrity error (“11”) while in the system the three types of data tampering attacks occur.

In theory, the security capability of LHash engine anticollision in instruction monitor can be represented as follows.

(3) $$P\left(m,n\right)=\frac{1}{C\left(m,n\right)\times 2^{n}}$$

where P(m,n) denotes the success probability for adversaries to guess correctly the digest value of BB integrity, in which *m* represents the digest size of LHash algorithm, and *n* represents the length of RNG-selected LHash bits from the *m* digest size. In our design, the success probability for an adversary to guess the correct integrity digest of each BB is $\frac{1}{C\left(96,16\right)\times 2^{16}}$, and this is almost impossible to achieve it during the period of each BB execution. In data protection, the 128 bits AES and 32 bits digital signature make it impossible for the attacker to reversely derive the desired plaintext and actualize tampering attack successfully in limited time, and this confidentiality method has a good robustness for resisting bus monitoring and offline analysis.

### 6.4. Comparison of Security and Practicality

In the routine protective measures, security and practicality are two most important metrics to evaluate hardware-assisted techniques. Our proposed SMU is a fully hardware-implemented unit, and it works at real-time without any modification on compiler and processor core, so that is easily transplanted to other embedded processors with different ISAs. Therefore, the comprehensive practicality is evaluated with the ISA extension, compiler modification, and performance overhead. Security capability is evaluated by the defense mechanisms against the instruction tampering, data tampering, and data leakage. We divided the security capability into the following four levels.

- Level-I: Only defend against instruction tampering or data tampering.
- Level-II: Both defend against data tampering and data leakage.
- Level-III: Both defend against instruction tampering and data leakage.
- Level-IV: Both defend against instruction tampering, data tampering, and data leakage.

As shown in Table 4, our proposed SMU achieves the instruction monitoring and data monitoring at a low (2.18%) performance overhead, and it is not necessary to extend ISAs and modify compilers. In addition, the SMU can achieve the level-IV without leaking dynamic data during data exchange with the main memory. Hence, the protection technology of SMU has a comprehensive security capability and is easier to implement than others.

## 7. Hardware Implementation Evaluation

This integrated SMU inevitably increases the SoC hardware overhead, chip area, and power consumption. The RTL-level architecture is synthesized, implemented, and verified on a Xilinx Virtex-5 FPGA platform. In addition, the Synopsys Design Compiler and ICC are used to synthesize the secure SoC into gate-level netlists and then place and route with SMIC 130-nm CMOS standard cell library. Table 5 shows the overall SoC hardware overhead on FPGA and the ASIC implementation on chip area and power consumption. The occupied slices of SMU is about 10.5% on the total SoC, and the BlockRAM is the most consumed storage resources for storing $INF_{ref}$ table and timestamps on FPGA. The hardware-friendly LHash engine with a Feistel-PG internal permutation structure requires 817 gate elements (GE), which is fewer than the another lightweight hash implementation of PHOTON [39], which internal permutation costs 1120 GE. In the ASIC of SoC, our SMU is a relatively smaller hardware module which occupies 36.4% of the chip area (larger than 10.5%) after the route due to the RAM library placements; meanwhile, its dynamic power keeps a low power consumption. The proposed SMU shows a good balance between security and practicality.

## 8. Conclusions

Instruction execution and data processing are the two important protective objects of embedded systems against various security threats, and the existing techniques make it hard to real-time monitor both instruction tampering, data tampering, and data leakage attacks simultaneously. This paper presents an embedded system by integrating an SMU for real-time guaranteeing instruction execution and data processing security. The hardware-assisted SMU architecture employs an instruction monitor to provide instruction integrity monitoring for preventing the malicious instruction tampering caused by the hardware Trojan or artificial modification. Meanwhile, the SMU also integrates a data monitor to provide the authenticated encryption for defending dynamic data leakages and data tampering attacks. Our proposed SMU is a comprehensive solution to enhance SoC program execution and data processing security against the diversiform off-chip attacks. The implementation results on the Virtex-5 FPGA platform show that the SMU can provide the high-efficiency monitoring for instruction and data while maintaining a low performance overhead. Its overhead ranged from 0.54% to 4.09% on typical 8-KB I/D-Caches. Moreover, the security capability evaluations show that the SMU can detect transfer and nontransfer instruction modifications and three types of data tampering attacks. Both instruction and data monitoring features incur minimal resource overhead and performance degradation. 

## Figures and Tables

**Table 1:** Performance overhead of SoC configured with SMU (8 KB I-Cache and 8 KB D-Cache).

Benchmark	Total Instr.	Total BB	Memory Size (KB)	I-Cache Hit	D-Cache Read Hit	D-Cache Write Hit	CPI without SMU	CPI with SMU	Perform. Overhead
AES	22,170	3535	13.81	99.23%	99.86%	99.73%	3.53	3.64	3.11%
basicmath	26,515	4327	16.90	98.18%	98.65%	98.62%	2.64	2.72	3.03%
bitcount	19,684	3344	13.06	97.98%	96.52%	96.48%	1.67	1.70	1.80%
blowfish	19,128	3247	12.68	97.75%	97.74%	97.67%	3.54	3.61	1.98%
CRC	18,941	3231	12.62	99.61%	98.41%	98.35%	1.72	1.75	1.74%
FFT	13,506	2143	8.37	96.29%	98.52%	98.41%	2.94	2.98	1.36%
OpenECC	56,313	6734	26.30	99.15%	99.17%	98.96%	3.18	3.31	4.09%
patricia	23,130	3853	15.05	97.68%	97.26%	97.05%	1.65	1.69	2.42%
quicksort	6707	1018	3.98	99.23%	99.10%	98.83%	1.86	1.87	0.54%
SHA1	20,455	3400	13.28	98.75%	99.38%	99.27%	2.35	2.39	1.70%
Average	–	–	13.61	98.39%	98.46%	98.34%	2.51	2.57	2.18%

**Table 2:** Performance overhead of a processor configured with different depths of the monitor cache.

Benchmark	CPI without SMU	CPI with SMU under the Different Depths of M-Cache					
		No M-Cache	Depth (16)	Depth (32)	Depth (64)	Depth (128)	Depth (256)
AES	3.53	5.16	4.52	3.98	3.78	3.65	3.64
basicmath	2.64	3.94	3.29	2.95	2.81	2.73	2.72
bitcount	1.67	2.37	2.15	1.87	1.79	1.71	1.70
blowfish	3.54	5.03	4.46	3.94	3.81	3.62	3.61
CRC	1.72	2.27	2.15	2.01	1.83	1.76	1.75
FFT	2.94	5.12	4.30	3.67	3.37	2.99	2.98
OpenECC	3.18	4.78	4.07	3.59	3.44	3.32	3.31
patricia	1.65	2.35	2.10	1.83	1.76	1.70	1.69
quicksort	1.86	2.64	2.38	2.08	1.89	1.87	1.87
SHA1	2.35	3.69	2.95	2.64	2.50	2.40	2.39
Average	2.51	3.74	3.24	2.86	2.60	2.58	2.57
Performance overhead	–	48.92%	29.07%	13.88%	3.59%	2.67%	2.18%

**Table 3:** Security capability tests of SoC with integrating SMU under different tampering attacks.

Tampering Attacks	Nontransfer Instr. Tampering	Transfer Instr. Tampering	Spoofing Attack	Relocation Attack	Replay Attack
Approaches	l.nop 0x0 l.nop 0x1	l.bf 1f730 l.bf 1f734	Write: 0x0000201E Read: 0x0000201E	Write: 0x00002016 Read: 0x00002012	T5: 32’h19d1458a T3: 32’h17a380b2
Instruction Tampering	15000000 15000001	13ff fffd 14000001	–	–	–
Data Tampering	–	–	32’h3a67e420 32’h3a67e000	32’h2b651d54 32’h467f57b2	32’h19d1458a 32’h17a380b2
Exception	LHash Error (“01”)	LHash Error (“01”) BB Absence (“10”)	Integrity Error (“11”)	Integrity Error (“11”)	Integrity Error (“11”)

**Table 4:** Comparison of security capability and comprehensive practicality.

Security Mechanism	Security Capability				Comprehensive Practicality		
	Level	Instruction Tampering	Data Tampering	Data Leakage	ISA Extension	Compiler Modification	Performance Overhead
HAM [33]	I	Yes	No	No	No	No	Medi (5.59%)
SM-FR [23]	I	Yes	No	No	No	No	High (9.33%)
HardRoot [34]	I	No	Yes	No	No	No	Low (2.80%)
KENALI [35]	I	No	Yes	No	No	No	High (7–15%)
FEDTIC [22]	II	No	Yes	Yes	No	No	Medi (7.60%)
InfoShield [36]	II	No	Yes	Yes	Yes	Yes	Low (<1.0%)
HEP [18]	II	No	Yes	Yes	No	No	Low (0.94%)
CFI-LEA [37]	III	Yes	No	Yes	Yes	Yes	Low (3.19%)
WLUD-NBL [9]	III	Yes	No	Yes	No	No	Low (3.60%)
CCFI [38]	III	Yes	No	Yes	Yes	Yes	High (52.0%)
Our SMU	IV	Yes	Yes	Yes	No	No	Low (2.18%)

**Table 5:** The SoC hardware overhead on FPGA and ASIC implementation.

Type	Resource Utilization	SoC	SMU
FPGA	Slice Registers	2674	1058
	Slice LUTs	17,836	2235
	Occupied Slices	7762	837
	BlockRAM/FIFO	58	53
ASIC	Chip Area	2.94 mm${}^{2}$	1.07 mm${}^{2}$
	Power Consumption	57.2 mW	7.9 mW

## References

[1] Lentaris G., Stratakos I., Stamoulias I., Soudris D., Lourakis M., Zabulis X. (2020). High-performance vision-based navigation on SoC FPGA for spacecraft proximity operations. IEEE Trans. Circuits Syst. Video Technol..

[2] Fayneh E., Yuffe M., Knoll E., Zelikson M., Abozaed M., Talker Y., Shmuely Z., Rahme S.A. 14 nm 6th-generation core processor SoC with low power consumption and improved performance. Proceedings of the IEEE International Solid-State Circuits Conference (ISSCC).

[3] Peña-Fernandez M., Lindoso A., Entrena L., Garcia-Valderas M., Morilla Y., Martín-Holgado P. (2019). Online error detection through trace infrastructure in ARM microprocessors. IEEE Trans. Nucl. Sci..

[4] Ray S., Peeters E., Tehranipoor M.M., Bhunia S. (2018). System-on-chip platform security assurance: Architecture and validation. Proc. IEEE.

[5] Basak A., Bhunia S., Tkacik T., Ray S. (2017). Security assurance for system-on-chip designs with untrusted IPs. IEEE Trans. Inf. Forensics Secur..

[6] Das S., Liu Y., Zhang W., Chandramohan M. (2016). Semantics-based online malware detection: Towards efficient real–time protection against malware. IEEE Trans. Inf. Forensics Secur..

[7] Chen Y., Sun W., Zhang N., Zheng Q., Lou W., Hou Y.T. (2019). Towards efficient fine-grained access control and trustworthy data processing for remote monitoring services in IoT. IEEE Trans. Inf. Forensics Secur..

[8] De A., Khan M.N.I., Nagarajan K., Ghosh S. (2020). HarTBleed: Using hardware Trojans for data leakage exploits. IEEE Trans. Very Large Scale Integr. Syst..

[9] Khan M.N.I., De A., Ghosh S. (2020). Cache-Out: Leaking cache memory using hardware Trojan. IEEE Trans. Very Large Scale Integr. Syst..

[10] Chen G., Jin H., Zou D., Zhou B.B., Liang Z., Zheng W., Shi X. (2013). Safestack: Automatically patching stack-based buffer overflow vulnerabilities. IEEE Trans. Dependable Secur. Comput..

[11] Alam M., Roy D.B., Bhattacharya S., Govindan V., Chakraborty R.S., Mukhopadhyay D. SmashClean: A hardware level mitigation to stack smashing attacks in OpenRISC. Proceedings of the ACM/IEEE International Conference on Formal Methods and Models for System Design (MEMOCODE).

[12] Zhang F., Kodituwakku H.A.D.E., Hines J.W., Coble J. (2019). Multilayer data-driven cyber-attack detection system for industrial control systems based on network, system, and process data. IEEE Trans. Ind. Inform..

[13] Bhunia S., Hsiao M.S., Banga M., Narasimhan S. (2014). Hardware Trojan attacks: Threat analysis and countermeasures. Proc. IEEE.

[14] Banga M., Hsiao M.S. A novel sustained vector technique for the detection of hardware Trojans. Proceedings of the 22nd International Conference on VLSI Design.

[15] Narasimhan S., Du D., Chakraborty R.S., Paul S., Wolff1 F., Papachristou C., Roy K., Bhunia S. Multiple-parameter side-channel analysis: A non-invasive hardware Trojan detection approach. Proceedings of the IEEE International Symposium on Hardware-Oriented Security and Trust (HOST).

[16] Koushanfar F., Mirhoseini A. (2011). A unified framework for multimodal submodular integrated circuits Trojan detection. IEEE Trans. Inf. Forensics Secur..

[17] Ghandali S., Moos T., Moradi A., Paar C. (2020). Side-channel hardware trojan for provably-secure SCA-protected implementations. IEEE Trans. Very Large Scale Integr. Syst..

[18] Wang W., Zhang X., Hao Q., Zhang Z., Xu B., Dong H., Xia T., Wang X. (2019). Hardware-enhanced protection for the runtime data security in embedded systems. Electronics.

[19] Younan Y., Joosen W., Piessens F. (2004). Code Injection in C and CPP: A Survey of Vulnerabilities and Countermeasures.

[20] Hoque T., Wang X., Basak A., Karam R., Bhunia S. Hardware Trojan attacks in embedded memory. Proceedings of the IEEE 36th VLSI Test Symposium (VTS).

[21] Nagarajan K., De A., Khan M., Ghosh S. (2020). Trapped: DRAM Trojan designs for information leakage and fault injection attacks. arXiv.

[22] Hong M., Guo H. (2010). FEDTIC: A security design for embedded systems with insecure external memory. Proceedings of the International Conference on Future Generation Information Technology.

[23] Wang X., Zhao Z., Xu D., Zhang Z., Hao Q., Liu M. (2020). An M-Cache-based security monitoring and fault recovery architecture for embedded processor. IEEE Trans. Very Large Scale Integr. Syst..

[24] Bresch C., Michelet A., Amato L., Meyer T., Hely D. A red team blue team approach towards a secure processor design with hardware shadow stack. Proceedings of the IEEE 2nd International Verification and Security Workshop (IVSW).

[25] Hu H., Shinde S., Adrian S., Chua Z.L., Saxena P., Liang Z. Data-oriented programming: On the expressiveness of non-control data attacks. Proceedings of the IEEE Symposium on Security and Privacy (SP).

[26] Wu W., Wu S., Zhang L., Zou J., Dong L. (2013). Lhash: A lightweight hash function. Proceedings of the International Conference on Information Security and Cryptology.

[27] Gupta S.S., Chattopadhyay A., Sinha K., Maitra S., Sinha B.P. (2013). High-performance hardware implementation for RC4 stream cipher. IEEE Trans. Comput..

[28] Wang G. (2010). An abuse-free fair contract-signing protocol based on the RSA signature. IEEE Trans. Inf. Forensics Secur..

[29] Kermani M.M., Masoleh A.R. (2012). Efficient and high-performance parallel hardware architectures for the AES-GCM. IEEE Trans. Comput..

[30] Wang X., Zhao Z., Xu D., Zhang Z., Hao Q., Liu M., Si Y. (2020). Two-stage checkpoint based security monitoring and fault recovery architecture for embedded processor. Electronics.

[31] Guthaus M.R., Ringenberg J.S., Ernst D., Austin T.M., Mudge T., Brown R.B. MiBench: A free, commercially representative embedded benchmark suite. Proceedings of the 4th Annual IEEE International Workshop Workload Characterization, WWC-4.

[32] Bakiri M., Titri S., Izeboudjen N., Abid F., Louiz F., Lazib D. Embedded system with Linux Kernel based on OpenRISC 1200-V3. Proceedings of the International Conference on Sciences of Electronics.

[33] Arora D., Ravi S., Raghunathan A., Jha N.K. (2006). Hardware-assisted run-time monitoring for secure program execution on embedded processors. IEEE Trans. Very Large Scale Integr. Syst..

[34] Tychalas D., Keliris A., Maniatakos M. (2020). Stealthy information leakage through peripheral exploitation in modern embedded systems. IEEE Trans. Device Mater. Reliab..

[35] Song C., Lee B., Lu K., Harris W.R., Kim T., Lee W. Enforcing kernel security invariants with data flow integrity. Proceedings of the 23th Annual Network and Distributed System Security Symposium.

[36] Shi W., Fryman J.B., Gu G., Lee H.S., Zhang Y., Yang J. InfoShield: A security architecture for protecting information usage in memory. Proceedings of the Twelfth International Symposium on High-Performance Computer Architecture.

[37] Qiu P., Lyu Y., Zhang J., Wang D., Qu G. (2018). Control flow integrity based on lightweight encryption architecture. IEEE Trans. Comput.-Aided Des. Integr. Circuits Syst..

[38] Mashtizadeh A.J., Bittau A., Boneh D., Mazières D. CCFI: Cryptographically enforced control flow integrity. Proceedings of the 22nd ACM SIGSAC Conference on Computer and Communications Security.

[39] Guo J., Peyrin T., Poschmann A. (2011). The PHOTON family of lightweight hash functions. Adv. Cryptol..

